# clustifyr: an R package for automated single-cell RNA sequencing cluster classification

**DOI:** 10.12688/f1000research.22969.2

**Published:** 2020-07-16

**Authors:** Rui Fu, Austin E. Gillen, Ryan M. Sheridan, Chengzhe Tian, Michelle Daya, Yue Hao, Jay R. Hesselberth, Kent A. Riemondy

**Affiliations:** 1 RNA Bioscience Initiative, University of Colorado School of Medicine, Aurora,, CO, 80045, USA; 2 Department of Biochemistry, University of Colorado Boulder, Boulder, CO, 80303, USA; 3 Biomedical Informatics & Personalized Medicine, University of Colorado Anschutz Medical Campus, Aurora, CO, 80045, USA; 4 Bioinformatics Research Center, North Carolina State University, Raleigh, NC, 27695, USA; 5 Department of Biochemistry and Molecular Genetics, University of Colorado School of Medicine, Aurora, CO, 80045, USA

**Keywords:** Single-cell RNA sequencing, cell type classification, gene expression profile, R package

## Abstract

Assignment of cell types from single-cell RNA sequencing (scRNA-seq) data remains a time-consuming and error-prone process. Current packages for identity assignment use limited types of reference data and often have rigid data structure requirements. We developed the clustifyr R package to leverage several external data types, including gene expression profiles to assign likely cell types using data from scRNA-seq, bulk RNA-seq, microarray expression data, or signature gene lists. We benchmark various parameters of a correlation-based approach and implement gene list enrichment methods. clustifyr is a lightweight and effective cell-type assignment tool developed for compatibility with various scRNA-seq analysis workflows. clustifyr is publicly available at https://github.com/rnabioco/clustifyr

## Introduction

Single-cell mRNA sequencing (scRNA-seq) promises to deliver elevated understanding of cellular mechanisms, cell heterogeneity within tissue, and developmental transitions ^1– 5^. A key challenge in scRNA-seq data analysis is the identification of cell types from single-cell transcriptomes. Manual inspection of the expression patterns from a small number of marker genes is still standard practice, which is both cumbersome and potentially inaccurate. Methods that compare cell type expression patterns against robust reference data provide additional confidence in cell type assignments and have the potential to automate and standardize cell type assignment. Unfortunately, current implementations of scRNA-seq suffer from several limitations ^3, 6, 7^ that further compound the problem of cell type identification. First, only RNA levels are measured, which may not correlate with cell surface marker or gene expression signatures identified through other experimental techniques. Second, due to the low capture rate of RNAs, low expressing genes may face detection problems regardless of sequencing depth. Many previously established markers of disease or developmental processes suffer from this issue, such as transcription factors. On the data analysis front, over or under-clustering can generate cluster markers that are uninformative for cell type labeling. In addition, cluster markers that are unrecognizable to an investigator may indicate potentially interesting unexpected cell types but can be very intimidating to interpret.

For these reasons, investigators struggle to integrate scRNA-seq into their studies due to the challenges of confidently identifying previously characterized or novel cell populations. Formalized data-driven approaches for assigning cell type labels to clusters greatly aid researchers in interrogating scRNA-seq experiments. Currently, multiple cell type assignment packages exist but they are specifically tailored towards input types or workflows ^8– 14^. Seurat, a popular toolkit for single cell RNA-seq analysis, implements a mutual nearest neighbor-based method to annotate cell types using another single cell RNA-seq dataset in the Seurat object format ^14^. SingleR and scmap provide functionality within the Bioconductor framework to annotate cell types using correlation if provided a reference from bulk-RNA-seq or averaged single cell cluster data ^8, 9^. scPred also uses a Bioconductor framework and applies a Support Vector Machine (SVM) model to PCA reduced gene expression data to classify cell types ^12^. ACTINN, a neural network-based annotation tool, also relies on existing single cell reference data and operates on files within a command line framework ^11^. As more and more approaches to the classification problem are introduced, benchmarking performance and compatibility to sequencing platforms and analysis pipelines becomes increasingly important.

We developed the R package clustifyr, a lightweight and flexible tool that leverages a wide range of prior knowledge of cell types to pinpoint target cells of interest or assign general cell identities to difficult-to-annotate clusters. Here, we demonstrate its basic usage and applications with transcriptomic information of external datasets and/or signature gene profiles, to explore and quantify likely cell types. The clustifyr package is built with compatibility and ease-of-use in mind to support other popular scRNA-seq tools and formats.

## Methods

### Implementation

**Figure 1.**
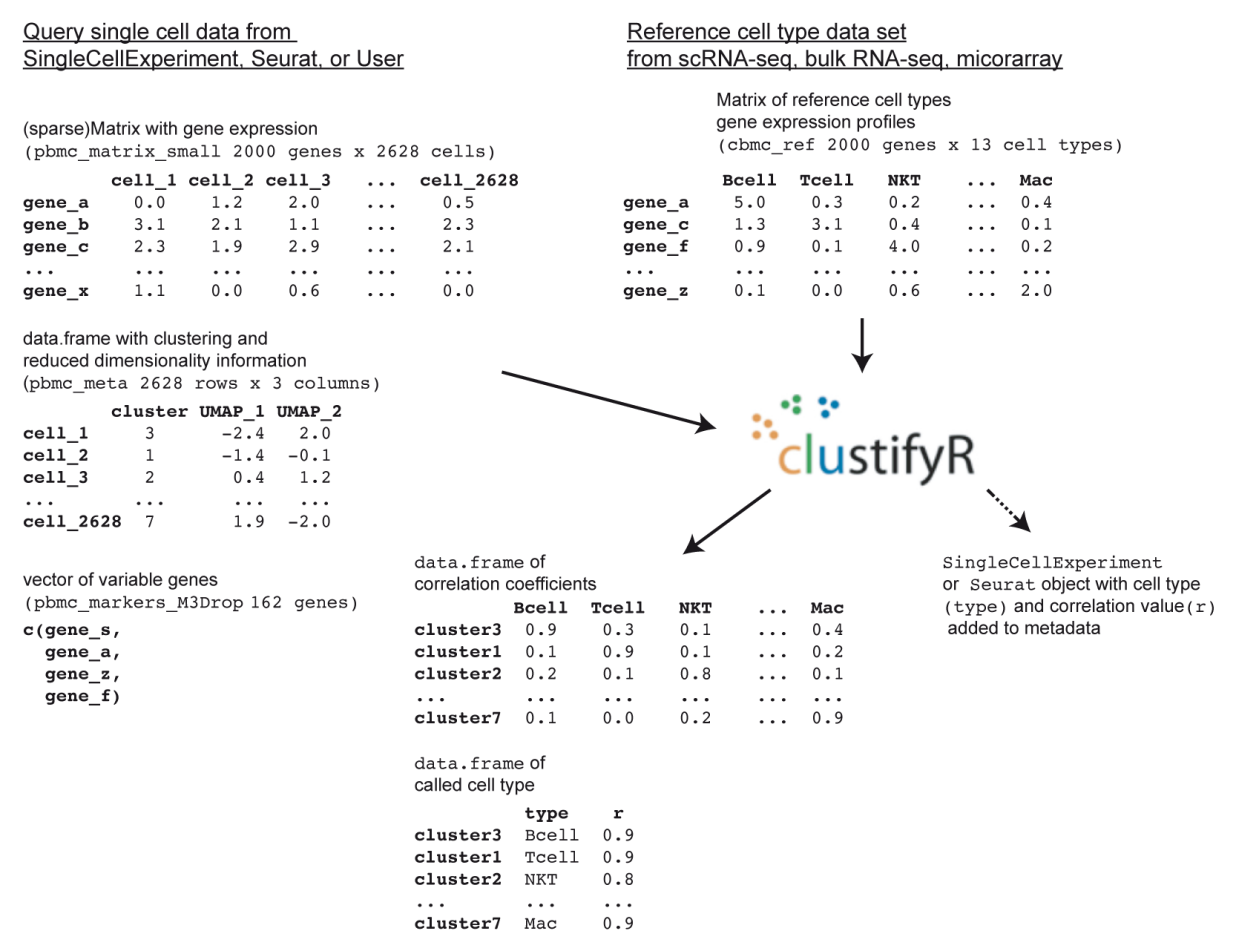
Schematic for clustifyr input and output.


clustifyr requires query and reference data in the form of normalized expression matrices, corresponding metadata tables, and a list of variable genes ( Figure 1).

library(clustifyr)
pbmc_matrix_small[1:5,1:5]# query matrix of normalized scRNA-
seq counts
cbmc_ref[1:5,1:5]# reference matrix of expression for each
cell type
pbmc_meta[1:5, ]# query meta-data data.frame containing cell
clusters
length(pbmc_markers_M3Drop$Gene)# vector of variable genes


clustifyr adopts correlation-based methods to find reference transcriptomes with the highest similarity to query cluster expression profiles, defaulting to Spearman ranked correlation, with options to use Pearson, Kendall, or Cosine correlation instead if desired. clustify() will return a matrix of correlation coefficients for each cell type and cluster, with the row names corresponding to the query cluster number and column names as the reference cell types.


res<-clustify(
input =pbmc_matrix_small,
metadata =pbmc_meta,
cluster_col ="seurat_clusters",# column in meta.data with
clusters
ref_mat =cbmc_ref,
query_genes =pbmc_markers_M3Drop
                        $Gene
)
res[1:5,1:5]
#>           B CD14+ Mono CD16+ Mono     CD34+     CD4 T
#> 0 0.4700038  0.5033242  0.5188112 0.6012423 0.7909705
#> 1 0.4850570  0.4900953  0.5232810 0.5884319 0.7366543
#> 2 0.5814309  0.9289886  0.8927613 0.6394140 0.5258430
#> 3 0.8609621  0.4663520  0.5686564 0.6429193 0.4698687
#> 4 0.2814882  0.1888232  0.2506101 0.4140560 0.6125503


Query clusters are assigned cell types to the highest correlated reference cell type, with an automatic or manual cutoff threshold. Query clusters dissimilar to all available reference cell types are labeled as “unassigned”.

res2<-cor_to_call(

cor_mat =res,# matrix of correlation coefficients
cluster_col ="seurat_clusters", # column in meta.data with
clusters

threshold =0.5
)



To better integrate with standard workflows that involve S3/S4 R objects, methods for clustifyr are written to directly recognize Seurat ^14^ (v2 and v3) and SingleCellExperiment ^15^ objects, retrieve the required information, and reinsert classification results back into an output object. A more general wrapper is also included for compatibility with other common data structures and can be easily extended to new object types. This approach also has the added benefit of forgoing certain calculations such as variable gene selection or clustering, which may already be stored within input objects.




res<-clustify(
input=sce_small,# an SCE object
ref_mat=cbmc_ref,# matrix of expression for each cell type
cluster_col="cell_type1",# column in meta.data with clusters
obj_out=TRUE# output SCE object with cell type
)
SingleCellExperiment::colData(res)[1:10, c("type","r")]
#> DataFrame with 10 rows and 2 columns
#>               type                 r
#>        <character>         <numeric>
#> AZ_A1         pDCs 0.814336567702192
#> AZ_A10       Eryth 0.665800619720566
#> AZ_A11        pDCs 0.682088309107356
#> AZ_A12       Eryth 0.665800619720566
#> AZ_A2            B 0.634114583333333
#> AZ_A3         pDCs 0.814336567702192
#> AZ_A4         pDCs 0.814336567702192
#> AZ_A5           NK 0.655407634437123
#> AZ_A6         pDCs 0.682088309107356
#> AZ_A7         pDCs  0.71424223704931



res<-clustify(

input=s_small3,# a Seurat object
ref_mat=cbmc_ref,# matrix of expression for each
cell type
  
  
                    cluster_col="RNA_snn_res.1",# name of column in meta.data
containing cell clusters
  
  
                    obj_out=TRUE# output Seurat object with cell
type inserted as "type" column

)

res@meta.data[1:5, ]

#>                   orig.ident nCount_RNA nFeature_RNA
RNA_snn_res.0.8

#> ATGCCAGAACGACT SeuratProject         70           47
0

#> CATGGCCTGTGCAT SeuratProject         85           52
0

#> GAACCTGATGAACC SeuratProject         87           50
1

#> TGACTGGATTCTCA SeuratProject        127           56
0

#> AGTCAGACTGCACA SeuratProject        173           53
0

#>                letter.idents groups RNA_snn_res.1 type
r

#> ATGCCAGAACGACT             A     g2             0   Mk
0.6204476

#> CATGGCCTGTGCAT             A     g1             0   Mk
0.6204476

#> GAACCTGATGAACC             B     g2             0   Mk
0.6204476

#> TGACTGGATTCTCA             A     g2             0   Mk
0.6204476

#> AGTCAGACTGCACA             A     g2             0   Mk
0.6204476

In the absence of suitable reference data (i.e. RNA-seq or microarray expression matrices), clustifyr can build scRNA-seq reference data by averaging per-cell expression data for each cluster, to generate a transcriptomic snapshot. Direct reference-building from SingleCellExperiment or Seurat objects is supported as well.


new_ref_matrix<-average_clusters(
mat=pbmc_matrix_small,
metadata=pbmc_meta$classified,# or use metadata = pbmc_meta, cluster_col = "classified"
if_log=TRUE# whether the expression matrix is already log transformed
)
new_ref_matrix_sce<-object_ref(
input=sce_small,# SCE object
cluster_col="cell_type1"# column in colData with cell identities
)

                        new_ref_matrix_v3<- seurat_ref(
seurat_object=s_small3,# SeuratV3 object
cluster_col="RNA_snn_res.1"# column in meta.data with cell identities
)


Data exploration plotting functions, for dimensional reduction scatter plots and heatmaps, are extended from ggplot2 and ComplexHeatmap packages, featuring colorblind-friendly default colors. Gene list-based methods (clustify_lists()) are also implemented via hypergeometric tests, GSEA, jaccard index, or percentage gene detection by cluster, which provide easy to interpret methods to verify the presence of known positive and negative marker genes.

### Parameters

***Reference datasets.*** Multiple scRNA-seq and other cell type references datasets are provided in an ExperimentHub Bioconductor package (clustifyrdatahub). A description of these datasets and others used for benchmarking and optimizing parameters for clustifyr are provided in Table 1.

**Table 1.** Collection of datasets used for introducing and benchmarking clustifyr. A description of single cell RNA-seq, bulk RNA-seq, and microarray datasets used in this study. The datasets available through ExperimentHub are references that were built from raw or downloaded data and can be used with clustifyr. R objects can be accessed using the direct download URLs to the .rda files, or through the clustifyrdatahub ExperimentHub.

Description	# of cell types	Organism	Publication	Source	Data Provider	R object download URL ^1^	Bioconductor ExperimentHubID ^2^	R object name ^3^
Mouse Cell Atlas	713	mouse	https://www.cell. com/cell/fulltext/S0092- 8674(18)30116-8	https://ndownloader.figshare.com/ files/10756795	figshare	https://github.com/ rnabioco/clustifyrdata/raw/ master/data/ref_MCA.rda	EH3444	ref_MCA
Tabula Muris (10X)	112	mouse	https://www.nature. com/articles/s41586- 018-0590-4	https://ndownloader.figshare.com/ articles/5821263	figshare	https://github.com/ rnabioco/clustifyrdata/raw/ master/data/ref_tabula_ muris_drop.rda	EH3445	ref_tabula_ muris_drop
Tabula Muris (SmartSeq2)	175	mouse	https://www.nature. com/articles/s41586- 018-0590-4	https://ndownloader.figshare.com/ articles/5821263	figshare	https://github.com/ rnabioco/clustifyrdata/raw/ master/data/ref_tabula_ muris_facs.rda	EH3446	ref_tabula_ muris_facs
Mouse RNA-seq from 28 cell types	28	mouse	https://genome. cshlp.org/content/ early/2019/03/11/ gr.240093.118	https://github.com/dviraran/ SingleR/tree/master/data	GitHub	https://github.com/ rnabioco/clustifyrdata/raw/ master/data/ref_mouse. rnaseq.rda	EH3447	ref_mouse. rnaseq
Mouse Organogenesis Cell Atlas (main cell types)	37	mouse	https://www.nature. com/articles/s41586- 019-0969-x	https://oncoscape.v3.sttrcancer. org/atlas.gs.washington.edu. mouse.rna/downloads	washington.edu	https://github.com/ rnabioco/clustifyrdata/raw/ master/data/ref_moca_ main.rda	EH3448	ref_moca_ main
Mouse sorted immune cells	253	mouse	https://www.nature. com/articles/ni1008- 1091	https://github.com/dviraran/ SingleR/tree/master/data	GitHub	https://github.com/ rnabioco/clustifyrdata/raw/ master/data/ref_immgen. rda	EH3449	ref_immgen
Human hematopoietic cell microarray	38	human	https://www.cell. com/fulltext/S0092- 8674(11)00005-5	https://ftp.ncbi.nlm.nih.gov/geo/ series/GSE24nnn/GSE24759/ matrix/GSE24759_series_matrix. txt.gz	GEO	https://github.com/ rnabioco/clustifyrdata/raw/ master/data/ref_hema_ microarray.rda	EH3450	ref_hema_ microarray
Human cortex development scRNA-seq	47	human	https://science. sciencemag.org/ content/358/6368/1318. long	https://cells.ucsc.edu/cortex-dev/ exprMatrix.tsv.gz	UCSC	https://github.com/ rnabioco/clustifyrdata/raw/ master/data/ref_cortex_ dev.rda	EH3451	ref_cortex_ dev
Human pancreatic cell scRNA-seq (inDrop)	14	human	https://www.cell. com/fulltext/S2405- 4712(16)30266-6	https://scrnaseq-public-datasets. s3.amazonaws.com/scater-objects/ baron-human.Rda	S3	https://github.com/ rnabioco/clustifyrdata/ raw/master/data/ref_pan_ indrop.rda	EH3452	ref_pan_ indrop
Human pancreatic cell scRNA-seq (SmartSeq2)	12	human	https://www. sciencedirect.com/ science/article/pii/ S1550413116304363	https://scrnaseq-public-datasets. s3.amazonaws.com/scater-objects/ segerstolpe.Rda	S3	https://github.com/ rnabioco/clustifyrdata/ raw/master/data/ref_pan_ smartseq2.rda	EH3453	ref_pan_ smartseq2
Human PBMCs, PBMC-Bench (multiple platforms)	9	human	https://doi.org/10.1186/ s13059-019-1795-z	https://zenodo.org/record/3357167/ files/scRNAseq_Benchmark_ datasets.zip?download=1	Zenodo	https://zenodo.org/ record/3357167/files/ scRNAseq_Benchmark_ datasets.zip?download=1	NA	NA
Human PBMCs, Unseen rejection test	5,7,10	human	https://doi.org/10.1186/ s13059-019-1795-z	https://zenodo.org/record/3357167/ files/scRNAseq_Benchmark_ datasets.zip?download=1	Zenodo	https://zenodo.org/ record/3357167/files/ scRNAseq_Benchmark_ datasets.zip?download=1	NA	NA
Mouse anterior lateral motor cortex (ALM)	34	mouse	https://doi.org/10.1038/ s41586-018-0654-5	https://portal.brain-map.org/ atlases-and-data/rnaseq/mouse- v1-and-alm-smart-seq	Allen Brain Institute	NA	NA	NA
Mouse brain primary visual cortex (VISp)	34	mouse	https://doi.org/10.1038/ s41586-018-0654-5	https://portal.brain-map.org/ atlases-and-data/rnaseq/mouse- v1-and-alm-smart-seq	Allen Brain Institute	NA	NA	NA
Human PBMC rejection test (SciBet)	5	human	https://doi.org/10.1038/ s41467-020-15523-2	http://scibet.cancer-pku.cn/ document.html	Investigator	NA	NA	NA
Human CBMC (CITE-Seq)	13	human	https://doi.org/10.1038/ nmeth.4380	ftp://ftp.ncbi.nlm.nih.gov/geo/ series/GSE100nnn/GSE100866/ suppl/GSE100866_CBMC_8K_ 13AB_10X-RNA_umi.csv.gz	GEO	NA	NA	NA
Human PBMCs (3k)	9	human	https://doi.org/10.1038/ ncomms14049	https://support.10xgenomics. com/single-cell-gene-expression/ datasets	10x Genomics	https://www.dropbox. com/s/63gnlw45jf7cje8/ pbmc3k_final.rds?dl=0	NA	NA

^1^download URL to access R object (if available) ^2^R object id in the clustifyrdatahub Bioconductor Experiment hub ^3^R object name (if available via clustifyrdatahub)

**Figure 2.**
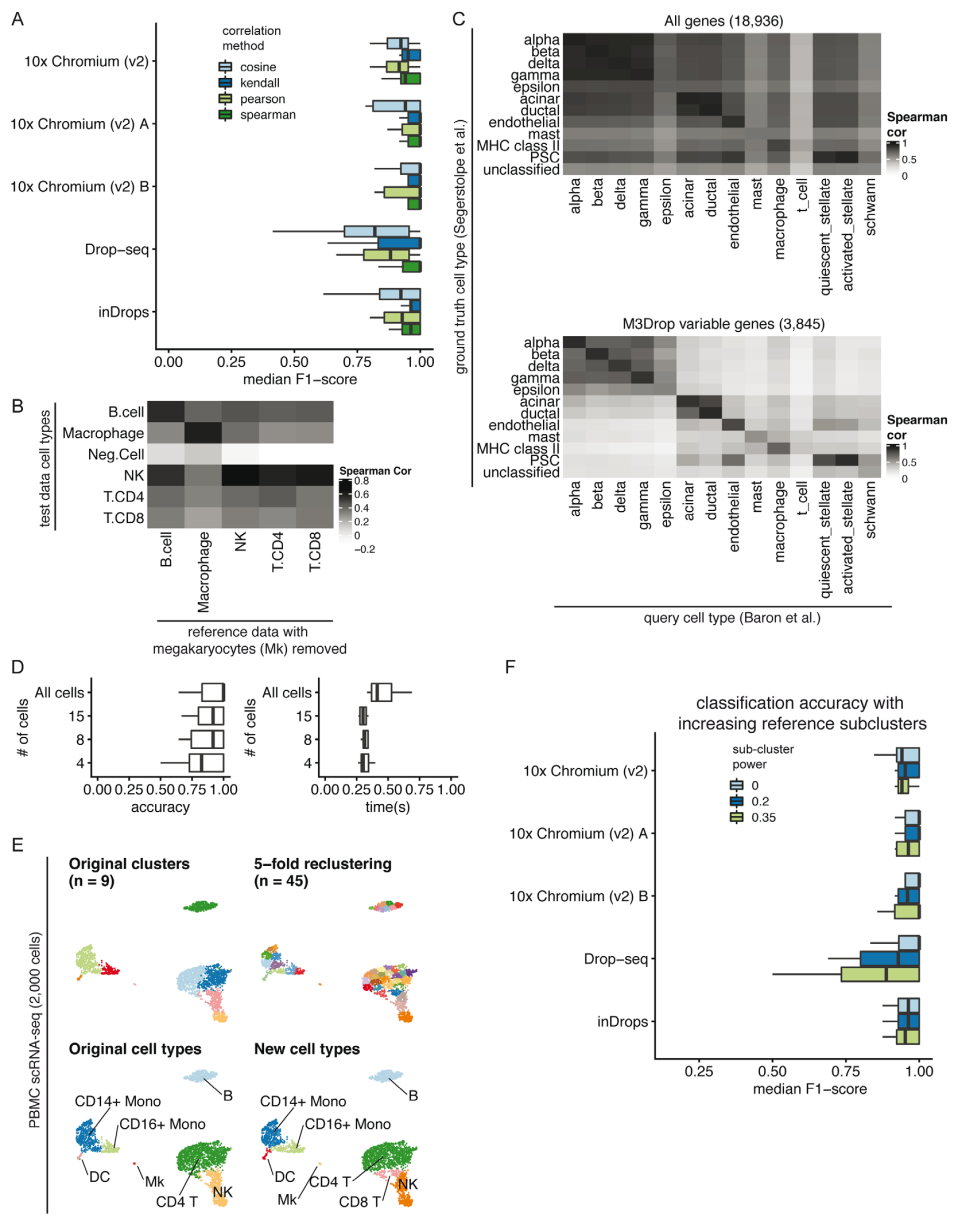
Parameter considerations for clustifyr. **A**) Comparison of median F1-scores of different correlation methods for classifying across platforms using the PBMC-bench dataset. **B**) Heatmap showing correlation coefficients between query cell types and the reference cell types from a rejection test, whereby megakaryocytes were excluded from the reference dataset. The Neg.Cell cluster is megakaryocytes, which is correctly not annotated a different cell type when megakaryocytes are not present in the reference. By default clusters with correlation < 0.50 are assigned as “unassigned” by clustifyr. **C**) Comparison of correlation coefficients with and without feature selection when comparing average gene expression per cell type between two pancreas scRNA-seq datasets. The “unclassified” cell type was not defined in the Segerstolpe *et al* dataset. **D**) Accuracy (defined as the ratio between the number of correctly classified clusters and the overall number of clusters) and performance were assessed with decreasing query cluster cell numbers using the Tabula Muris as the query dataset and the Mouse cell atlas as the reference dataset. **E**) Example of overclustering the query data and assigning cell types for data exploration. UMAP of PBMC dataset generated by 10x Genomics with cell types assigned by comparing to reference data from CBMC cells from Stoeckius *et al.* 2017. **F**) An assessment of the median F1-score when using single or multiple averaged profiles as reference cell types was conducted using the PBMC-bench test set. The number of reference expression profiles to generate for each cell type is determined by the number of cells in the cluster (n), and the sub-clustering power argument (x), with the formula n ^x^.

***Correlation method.*** We benchmarked clustifyr against a suite of comparable datasets, PBMC-bench ^13, 16^, generated using multiple scRNA-seq methods on aliquots of peripheral blood mononuclear cells (PBMCs) from two individuals. Additional details about each query and reference dataset are provided in Supplemental Table 1. For each single cell technology, average gene expression profiles were generated from annotated cell types and compared across each platform. Notably, for each reference dataset cross-referenced against all other samples, clustifyr achieved a median F1-score (see Benchmarking Methods) of above 0.94 using Spearman ranked correlation ( Figure 2A). Other correlation methods are on par or slightly worse at cross-platform classifications, which is expected based on the nature of ranked vs unranked methods. We therefore selected Spearman as the default method in clustifyr, with other methods also available, as well as a wrapper function to find consensus identities across available correlation methods (call_consensus()).

***Correlation minimum cutoff.*** Recognition of missing reference cell types, so as to avoid misclassification, is another point of great interest in the field. From general usage of clustifyr, we find using a minimum correlation cutoff of 0.5 or 0.4 is generally satisfactory. Alternatively, the cutoff threshold can be determined heuristically using 0.8 * highest correlation coefficient among the clusters. One example is shown in Figure 2B, using PBMC rejection benchmark data modified by the SciBet package ^17^. Megakaryocytes were removed from the reference melanoma immune cells data, but retained in the test data to mimic the situation when the reference data does not contain a rare cell type. clustifyr analysis successfully found the megakaryocytes to be dissimilar to all available reference cell types, and hence left as “unassigned” under the default minimum threshold cutoff.

***Variable gene selection and normalization.*** As the core function of clustifyr is ranked correlation, feature selection to focus on highly variable genes is critical. To illustrate the importance of feature selection we used clustifyr to classify pancreatic cell types generated using the inDrops platform using a reference built from a dataset generated on the Smart-Seq2 platform ^18, 19^. In Figure 2C, we compare correlation coefficients using all detected genes (>10,000) vs feature selection by the package M3Drop. A basic level of feature selection, e.g. using M3Drop, Seurat VST (default uses top 2,000 variable genes), or simply 1,000 genes with the highest variance in the reference data, is sufficient to classify the pancreatic cells. In the case of other cell type mixtures, especially ones without complete knowledge of the expected cell types, further optimization of clustering and feature selection may be of greater importance. clustifyr does not provide novel clustering, feature selection, or normalization methods on its own, but instead is built to maintain flexibility to incorporate methods from other, and future, packages. We recommend that users use normalized reference and query data and match normalization methods between datasets when possible. We view these questions as fast-moving fields ^20, 21^, and hope to benefit from new advances, while keeping the general clustifyr framework intact.

***Minimum cells per cluster.*** We next applied clustifyr to a larger general reference set built from the Mouse Cell Atlas ^22^ and examined cell type classification of another mouse cell atlas, the Tabula Muris dataset ^5^. clustifyr assigned cell types with a median accuracy of 1. Using these test datasets we sought to determine the minimum number of query cells necessary in a cluster to obtain accurate cell type annotation. We subsampled the query data ( Figure 2D) and as expected, with further downsampling of the number of cells in each query cluster, we observe decreased accuracy. Yet, even at 15 cells per tested cluster, clustifyr still performed well, with a further increase in speed. Based on these results, we set the default parameters in clustifyr to exclude or warn users of classification on clusters containing less than 10 cells. These results also suggest that clustering the query dataset to obtain more refined clusters (e.g fewer cells per cluster) could be employed to aid in the identification of rarer or less well-defined cell subsets. clustifyr can also be used to classify individual cells, although we do not recommend per cell classification because of the reduced accuracy observed with decreasing numbers of cells per cluster.

***Subclustering.*** clustifyr also provides functionality to assess the quality of the cell type annotations. An intentional overclustering and classification function based on k-means clustering (overcluster_test()) is implemented in clustifyr for exploration of cell type annotation at increasing numbers of clusters ( Figure 2E). This approach provides a rapid visualization to determine if cell type annotations are stable with varying numbers of clusters. For example, scRNA-seq data from the Seurat PBMC 3k tutorial was reclassified at multiple clustering levels using Cord Blood Mononuclear Cells (CBMCs) as reference, which demonstrated largely stable cell type assignments in the presence of overclustered query data ( Figure 2E) ^23^. When using scRNA-seq data as the reference data, matrices are built by averaging per-cell expression data for each cluster (average_clusters()), to generate a transcriptomic snapshot similar to bulk RNA-seq or microarray data. An additional argument to subcluster the reference single cell clusters is also available, to generate more than one expression profile per reference cell type, in a manner analogous to overcluster_test(), but applied to the reference scRNA-seq dataset. The number of subclusters for each reference cell type is dependent on the number of cells in the cluster (n), and the sub-clustering power argument (x), following the formula n ^x^ ^9^. This approach does not improve classification in the PBMC-bench data ( Figure 2F), whose reference and query clustering are already consistent. However, we envision its utility would greatly depend on the granularity of the clustering in the reference dataset.

### Benchmarking

**Figure 3.**
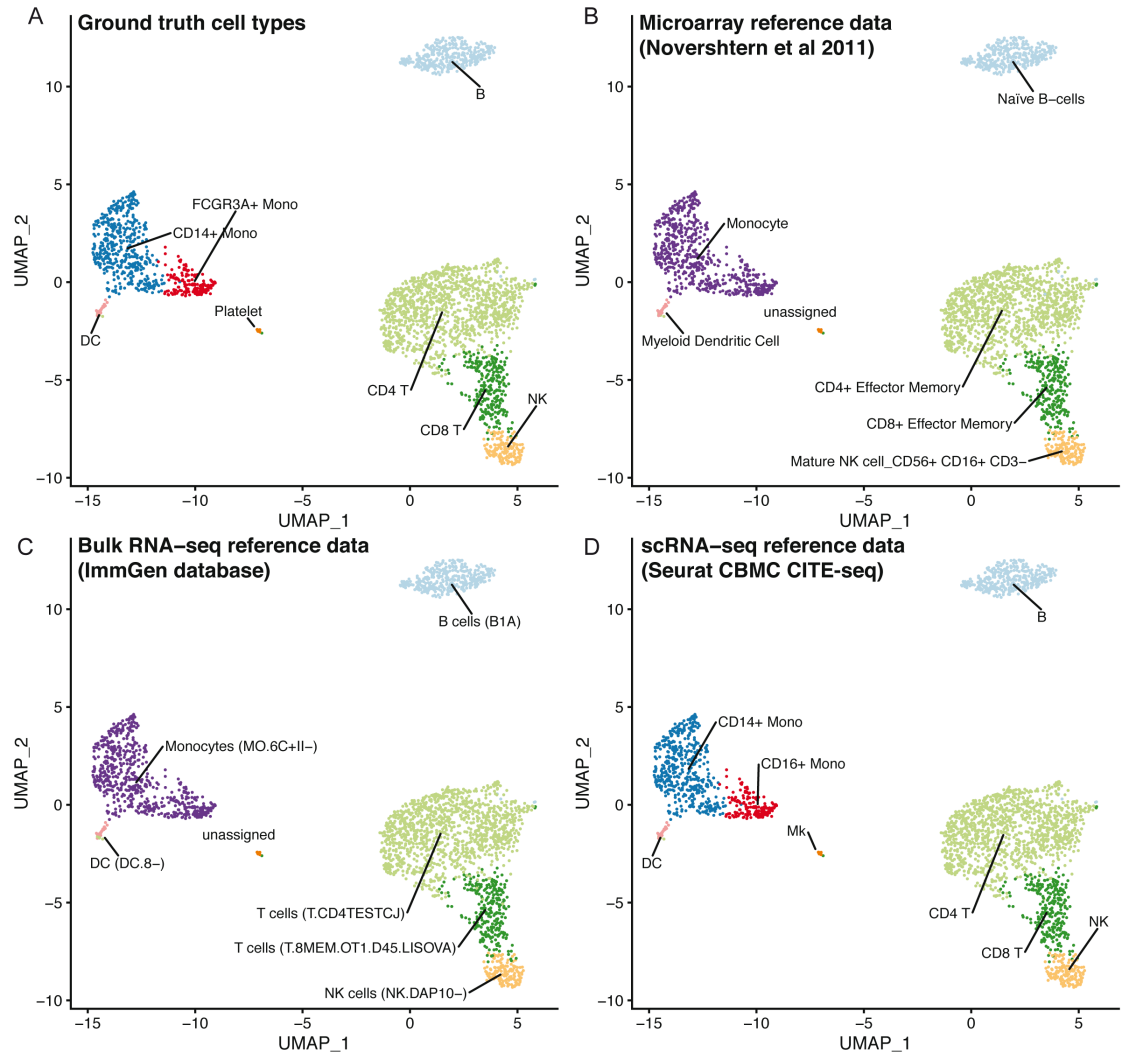
clustifyr can utilize multiple reference data types. UMAP projections of PBMCs showing the ground truth cell types ( **A**), or cell types called by clustifyr using microarray data from sorted immune cell types ( **B**), bulk RNA-seq from immune cell populations ( **C**) or scRNA-seq data from CBMCs ( **D**).

Using clustifyr, PBMC clusters from the Seurat PBMC 3k tutorial are correctly labeled using either bulk-RNA seq references generated from processed microarray data of purified cell types ^24^, the ImmGen database of bulk-RNA-seq ^9, 25^, or previously annotated scRNA-seq results from the Seurat CBMC CITE-seq tutorial ^14, 23^ ( Figure 3).

To assess the performance of clustifyr, we used the Tabula Muris dataset ^5^, which contains data generated from 12 matching tissues using both 10x Genomics 3’ end seq (“drop”) and Smart-Seq2 (“facs”) platforms. We attempted to assign cell type identities to clusters in “drop” Seurat objects using references built from “facs” Seurat objects, which contain pre-computed variable genes generated by the Seurat mean.var.plot (dispersion z-scores based on expression bins) approach. For each method we used the recommended variable gene selection approach. clustifyr uses variable genes supplied by the user and for benchmarking we used the variable genes stored in the Seurat object. scmap calculates variable genes using a modified approach based on M3drop. SingleR selects variable genes by identifying marker genes between clusters. scPred, in contrast, selects informative principal components as a feature selection procedure whereas ACTINN does not perform feature selection for classification.

**Figure 4.**
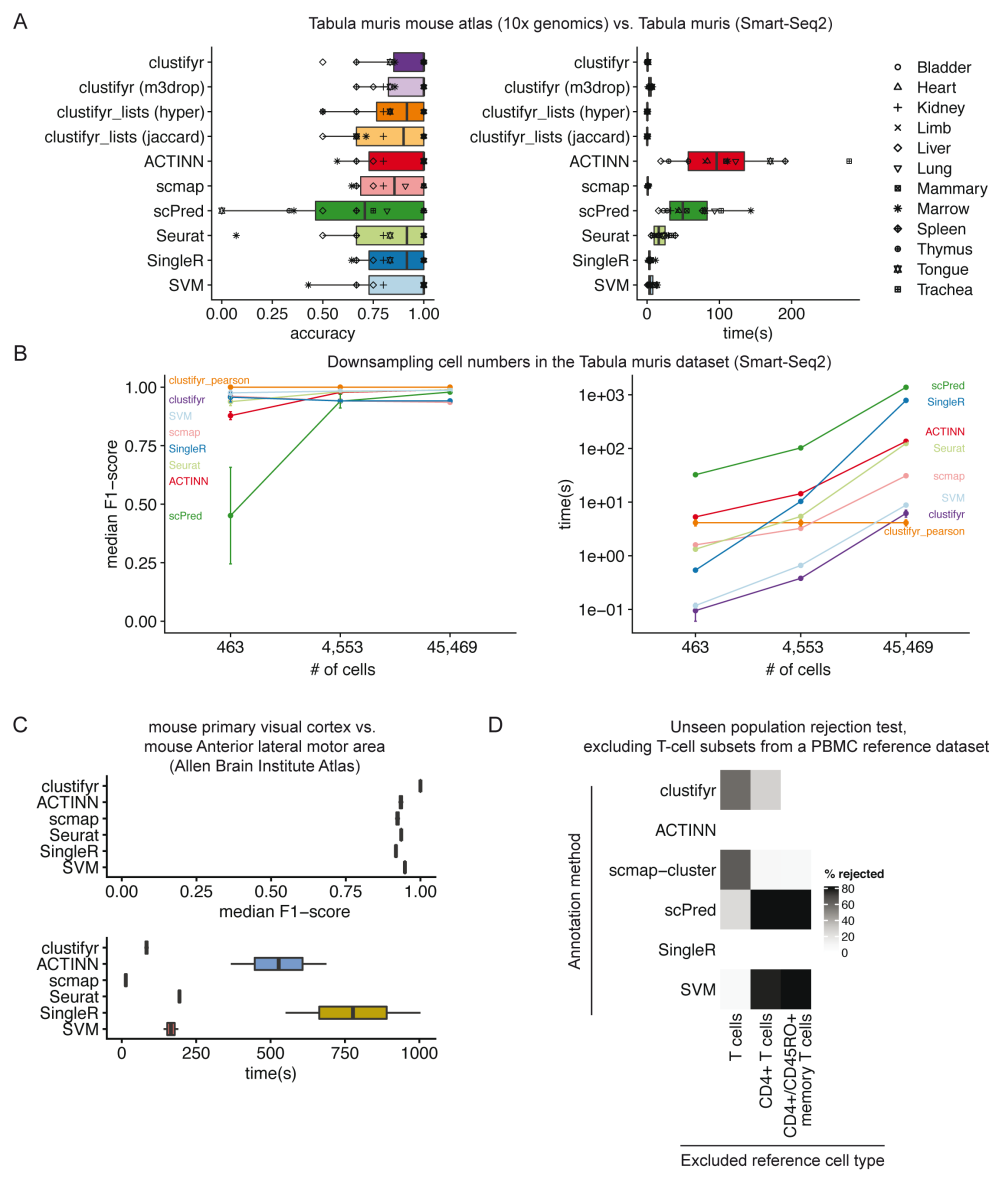
clustifyr accurately and rapidly annotates cell types. **A**) Accuracy and run-time of classifications generated by clustifyr or existing methods using the Tabula Muris dataset to benchmark cell type classifications between datasets generated with the Smart-Seq2 or 10x Genomics sequencing platforms. Each point represents a different tissue comparison. clustifyr (m3drop) indicates clustifyr run using variable genes defined by M3drop, clustifyr_lists (hyper) uses hypergeometric tests to compare marker gene lists, and clustifyr_lists(jaccard) calculates the jaccard index between marker gene lists to annotate cell types. **B**) Performance comparison of clustifyr to existing methods with random subsamples of cells from the Smart-Seq2 Tabula Muris dataset. Error bars represent standard error of the mean and are derived from 5 independent subsamples of the dataset. **C**) Performance comparison of clustifyr to existing methods testing classification of an Allen Institute Brain Atlas dataset from two murine brain regions that contain 34 cell types. scPred is not shown as it failed with an error on this dataset. **D**) Comparing clustifyr to existing methods for rejecting unseen populations using PBMC data. Three reference PBMC datasets were generated that excluded either T-cells, CD4+ T-cells or memory T-cells respectively. The % of rejected indicates the % of the indicated cell type that was not misclassified when the cell type was missing from the reference.

 In benchmarking results, clustifyr is comparably accurate versus other automated classification packages ( Figure 4A). Cross-platform comparisons are inherently more difficult, and the approach used by clustifyr is aimed at being platform- and normalization-agnostic. Mean runtime, including both reference building and test data classification, in Tabular Muris classifications was ~ 1 second if the required variable gene list is extracted from the query Seurat object. Alternatively, variable genes can be recalculated by other methods such as M3Drop ^26^, to reach similar results (clustifyr (m3drop)).

Signature marker gene lists are an additional reference data type that is commonly used to guide cluster cell type classification. We therefore sought to determine if a gene list enrichment approach could provide comparable classification power to using correlation. clustifyr provides a function clustify_lists() which compares marker genes between query clusters to a list of marker genes per reference cell type. clustify_lists will calculate enrichment with a hypergeometric test, marker overlap with the jaccard index, or use the percent of cells expressing marker genes to annotate cell types. Alternatively, if ranked gene lists are available, Gene Set Enrichment Analysis (GSEA) using the fGSEA package ^27^ or Spearman ranked correlation can be employed. We find that using gene expression for clustifyr classification had higher accuracy than gene list enrichment using a hypergeometric test or the jaccard index, however this approach could be very useful for datasets without scRNA or bulk RNA-seq data for use as a reference. ( Figure 4A).

For scalability benchmarking, we adapted an existing benchmark dataset, scRNAseq_Benchmark subsampling, which contains query and reference data with downsampled numbers of cells from the Smart-seq2 Tabula Muris dataset ^5, 13^. Once again, clustifyr is accurate and efficient, compared to other developed methods ( Figure 4B). As a further comparison, we also examined classification of cell types in murine brain datasets generated by the Allen Institute Brain Atlas, and provided by the scRNAseq_Benchmark pipeline ^13^. The two murine brain regions contained 34 shared cell types and clustifyr was also able to reach similarly satisfactory cell annotation compared to other annotation methods. ( Figure 4C).

Lastly, we applied clustifyr to a series of increasingly challenging datasets from the scRNAseq_Benchmark ^13^ unseen population rejection test ( Figure 4D). This test assess how frequently cells will be mis-assigned when the corresponded cell types are not present in the dataset. The PBMC dataset contains different T-cell subsets, which do not often cluster into discrete well-defined cell types solely based on gene expression. Without the corresponding cell type references, 57.5% of T cells were rejected and unassigned. When only CD4+ references were removed, 28.2% of test CD4+ T cells were rejected and unassigned. clustifyr was unable to reject CD4+/CD45RO+ memory T cells, mislabeling them as CD4+/CD25 T Reg instead when the exact reference was unavailable. However, these misclassifications are also observed with other classification tools benchmarked in the scRNAseq_Benchmark study ( Figure 4D) ^13^.

### Benchmarking methods

clustifyr was tested against scmap v1.8.0 ^8^, SingleR v1.0.1 ^9^, Seurat v3.1.1 ^14^, latest GitHub versions of ACTINN ^11^ and scPred ^12^, and SVM as implemented in python3 scikit-learn v0.19.1 ^28^. scRNA-seq Tabula Muris data was downloaded as seuratV2 objects. Human pancreas data was downloaded as SCE objects. In all instances, to mimic the usage case of clustifyr, clustering and dimension reduction projections are acquired from available metadata, in lieu of new analysis.

An R script was modified to benchmark clustifyr following the approach and datasets of scRNAseq_Benchmark ^13^, using M3Drop ^26^ to generate variable genes for clustifyr. R code used for benchmarking, and preprocessing of other datasets, in the form of matrices and tables, are documented in R scripts available in the clustifyr and clustifyrdatahub GitHub repositories.

Classification accuracy was measured using two approaches depending on the datasets compared. For datasets where the query and reference data contain identical cell types, an F1-score, the harmonic mean of the precision and recall, was calculated for each cell type (PBMC-bench, Allen Brain Institute Atlas, and Smart-Seq2 Tabula Muris subsampling). When summarizing classification accuracy across an entire dataset the median F1-score is reported. Datasets with varying cell types in the query and reference data cannot be characterized with an F1-score and instead accuracy, defined as the ratio between the number of correctly classified clusters and the overall number of clusters, is reported (Mouse cell atlas vs. Tabula Muris and Tabula Muris Smart-Seq2 vs. 10x Genomics).

### Operation

clustifyr is distributed as part of the Bioconductor R package repository and is compatible with Mac OS X, Windows, and major Linux operating systems. Package dependencies and system requirements are documented in the clustifyr Bioconductor repository.

## Conclusions

We present a flexible and lightweight R package for cluster identity assignment. The tool bridges various forms of prior knowledge and scRNA-seq analysis. Reference sources can include scRNA-seq data with cell types assigned (or average expression per cell type, which can be stored at much smaller file sizes), sorted bulk RNA-seq, and microarray data. clustifyr, with minimal package dependencies, is compatible with a number of standard analysis workflows such as Seurat or Bioconductor, without requiring the user to perform the error-prone process of converting to a new scRNA-seq data structure and can be easily extended to incorporate other data storage object types. clustifyr is designed to perform classification after previous steps of analysis by other informatics tools. Therefore, it relies on, and is agnostic to, common external packages for cell clustering and variable feature selection. We envision it to be compatible with all current and future scRNA-seq processing, clustering, and marker gene discovery workflows. Benchmarking reveals the package performs well in mapping cluster identity across different scRNA-seq platforms and experimental types. As we and others observe ^29^, novel algorithms may not be necessary for cell type classification, at least within the current limitations of sequencing technology and our broadstroke understanding of cell “types”. Rather, the generation of community curated reference databases is likely to be critical for reproducible annotation of cell types in scRNA-seq datasets.

On the user end, clustifyr is built with simple out-of-the-box wrapper functions, sensible defaults, yet also extensive options for more experienced users. Instead of building an additional single-cell-specific data structure, or requiring specific scRNA-seq pipeline packages, it simply handles basic data.frames (tables) and matrices ( Figure 1). Input query data and reference data are intentionally kept in expression matrix form for maximum flexibility, ease-of-use, and ease-of-interpretation. Also, by operating on predefined clusters, clustifyr has high scalability and minimal resource requirements on large datasets. Using per-cluster expression averages results in rapid classification. However, cell-type annotation accuracy is therefore heavily reliant on appropriate selection of the number of clusters. Users are therefore encouraged to explore cell type annotations derived from multiple clustering settings. Additionally, assigning cell types using discrete clusters may not be appropriate for datasets with continuous cellular transitions such as developmental processes, which are more suited to trajectory inference analysis methods. As an alternative, clustifyr also supports per-cell annotation, however the runtime is greatly increased and the accuracy of the cell type classifications are decreased due to the sparsity of scRNA-seq datasets, and requires a consensus aggregation step across multiple cells to obtain reliable cell type annotations.

To further improve the user experience, clustifyr provides easy-to-extend implementations to identify and extract data from established scRNA-seq object formats, such as Seurat ^14^, SingleCellExperiment ^15^, URD ^4^, and CellDataSet (Monocle) ^30^. Available in flexible wrapper functions, both reference building and new classification can be directly achieved through scRNA-seq objects at hand, without going through format conversions or manual extraction. The wrappers can also be expanded to other single cell RNA-seq object types, including the HDF5-backed loom objects, as well as other data types generated by CITE-seq and similar experiments ^31^. Tutorials are documented online to help users integrate clustifyr into their workflows with these and other bioinformatics software.

## Software availability

clustifyr is available from Bioconductor: https://bioconductor.org/packages/release/bioc/html/clustifyr.html

Up-to-date source code, and tutorials are available from: https://github.com/rnabioco/clustifyr

Package documentation is also provided at: https://rnabioco.github.io/clustifyr/

Archived source code as at time of publication and Supplemental Table 1 detailing datasets used in each analysis are available from:

https://doi.org/10.5281/zenodo.3934480 ^32^

Data used in examples and additional prebuilt references available from: https://github.com/rnabioco/clustifyrdatahub

License: MIT

## Data availability

Original raw data used in benchmarking is available from the following sources and additionally described in Table 1.

**Table:** 

Dataset	Source
PBMC 3k Seurat V3 object	https://www.dropbox.com/s/63gnlw45jf7cje8/pbmc3k_final.rds?dl=0
CBMC CITE-seq	Accession number, GSE100866: ftp://ftp.ncbi.nlm.nih.gov/geo/series/GSE100nnn/ GSE100866/suppl/GSE100866_CBMC_8K_13AB_10X-RNA_umi.csv.gz
Hematopoiesis microarray data	Accession number, GSE24759: https://www.ncbi.nlm.nih.gov/geo/query/acc.cgi?acc=GSE24759
Tabula Muris as Seurat V2 objects	https://figshare.com/projects/Tabula_Muris_Transcriptomic_characterization_of_20_organs_ and_tissues_from_Mus_musculus_at_single_cell_resolution/27733
Mouse Cell Atlas	https://doi.org/10.6084/m9.figshare.5435866.v8
Pancreatic scRNA-seq as SingleCellExperiment objects	https://hemberg-lab.github.io/scRNA.seq.datasets/
Allen Institute Brain Atlas	http://celltypes.brain-map.org/rnaseq
PBMC-bench	https://singlecell.broadinstitute.org/single_cell/study/SCP424/single-cell-comparison-pbmc-data
PBMC rejection test	http://scibet.cancer-pku.cn/document.html
ImmGen Database	http://www.immgen.org/
