# Supplementary material for: clustifyr: an R package for automated single-cell RNA sequencing cluster classification
Source: F1000Res. 2020 Jul 16;9:223. Originally published 2020 Apr 1. [Version 2] doi: 10.12688/f1000research.22969.2 (PMC7383722; doi:10.12688/f1000research.22969.2)
Supplement: Peer review history

# Peer review: Reviewer response for version 2

**DOI:** 10.5256/f1000research.27827.r67326

**Published:** 2020-07-24

**Authors:** Kamil Slowikowski

**Affiliations:** 1 Massachusetts General Hospital, Boston, MA, USA

Thank you for improving the manuscript!

 I have no further comments.

Are the conclusions about the tool and its performance adequately supported by the findings presented in the article?

Partly

Is the rationale for developing the new software tool clearly explained?

Yes

Is the description of the software tool technically sound?

Yes

Are sufficient details of the code, methods and analysis (if applicable) provided to allow replication of the software development and its use by others?

Partly

Is sufficient information provided to allow interpretation of the expected output datasets and any results generated using the tool?

Yes

Reviewer Expertise:

Bioinformatics, computational biology, immunogenomics, scRNA-seq.

I confirm that I have read this submission and believe that I have an appropriate level of expertise to confirm that it is of an acceptable scientific standard.

# Peer review: Reviewer response for version 2

**DOI:** 10.5256/f1000research.27827.r67325

**Published:** 2020-07-17

**Authors:** Keegan Korthauer

**Affiliations:** 1 BC Children's Hospital Research Institute, Vancouver, BC, Canada; 2 University of British Columbia, Vancouver, BC, Canada

The authors have addressed all comments and concerns.

Are the conclusions about the tool and its performance adequately supported by the findings presented in the article?

Partly

Is the rationale for developing the new software tool clearly explained?

Yes

Is the description of the software tool technically sound?

Yes

Are sufficient details of the code, methods and analysis (if applicable) provided to allow replication of the software development and its use by others?

Yes

Is sufficient information provided to allow interpretation of the expected output datasets and any results generated using the tool?

Partly

Reviewer Expertise:

Statistical genomics, bioinformatics

I confirm that I have read this submission and believe that I have an appropriate level of expertise to confirm that it is of an acceptable scientific standard.

# Peer review: Reviewer response for version 1

**DOI:** 10.5256/f1000research.25358.r63065

**Published:** 2020-06-02

**Authors:** Kamil Slowikowski

**Affiliations:** 1 Massachusetts General Hospital, Boston, MA, USA

The authors describe an R package for annotating cell clusters in scRNA-seq datasets. Specifically, the package implements code for computing correlations between the columns of two data matrices. They show that high correlations between unknown cell clusters in the first data matrix and annotated cell types in the second matrix can be used to label the unknown cell clusters. They try varying parameters and show the effects on the results, and they also benchmark the time and accuracy compared to other packages designed to annotate scRNA-seq data.

 Details of the code, methods, and analyses are partly provided. Some details seem to be missing (e.g. the functionality for gene lists).

 The conclusions about the tool and its performance are partly supported by the findings presented in the article. Some terms such as "medF1-score" and "accuracy" are left undefined, and some results omit some methods (Figure 4A has different methods than B or C). Readers may have difficulty understanding the specific questions that were asked and what results are shown.

 Main comments:

1. The clarity of the manuscript can be increased by adding more verbose details about all analyses. Please consider expanding details about each question, the approach, the datasets used, and the results.
2. Please consider adding a table describing the reference datasets used in this article, just like the one shown on one of your GitHub repositories. This should help to summarize which datasets were used for the analyses in this article.

Comments about specific parts of the manuscript are below. Excerpts from the article are shown in *"quoted* *italics"* after a bullet point, and my comments are shown directly below the bullet point.

  

- *"A key challenge in scRNA-seq data analysis is the identification of cell types from single-cell transcriptomes. Manual inspection of the expression patterns from a small number of marker genes is still standard practice, which is cumbersome and frequently inaccurate."*

Do we know the accuracy by manual inspection? Is there a reference for this? In the absence of evidence, you might consider weakening the statement to say "may be inaccurate" rather than "is cumbersome and frequently inaccurate". You might consider that many scRNA-seq experiments are done for the purpose of discovering new cell types that have not been well-described in previous published datasets. In this setting, manual inspection is necessary and automated analyses could be inaccurate or misleading.

  

- *"Currently, multiple cell type assignment packages exist but they are specifically tailored towards input types or workflows ^8–13^."*

Please consider naming and describing each method that will be compared to clustifyr in this manuscript, so the reader can assess how the methodology of clustifyr compares to other methods. Which methods are "specifically tailored towards input types or workflows"? Could you give an example to help the reader understand this claim?

**Suggested improvements for Figure 1:**

- In Figure 1, you might consider showing the dimensions of the inputs and outputs. This might help the reader to understand how they relate to each other.
- Should the query and reference data be counts? CPM? Or Log2(CPM + 1)? You might consider elaborating on this.

**Suggested improvements for Figure 2:**

- Please consider rotating Figure 2A, D, and E 90 degrees clockwise to improve legibility.
- Please consider limiting the axes ranges to the data instead of using the range [0, 1].
- Please consider increasing all font sizes in all panels in all figures, including titles, legends, axis text, etc. Some readers might need larger sizes to see clearly.
- Please consider changing the title to "All genes (n = 10,000)" and "M3Drop variable genes (n = 1,000)"  in Figure 2C, so we have some sense of the number of genes used to generate each heatmap.
- Please consider showing a graphical representation of the experiment setup for this figure. What is the reference? What is the query? What are their dimensions? What is the main question in this analysis?
- One way to enhance clarity is to add descriptive titles to every figure in every panel (e.g. "Testing different correlation statistics", etc.).
- Please consider adding more details to the legend text for Figure 2 to help readers understand exactly what experiment has been done, what data was used, and what result is shown.
- In Figure 2C, it seems that the y-axis and x-axis have been swapped by mistake. I see that the y-axis is labeled "ground truth cell type" but it includes "unclassified". I would expect the category "unclassified" to appear in the "called cell type" axis, but not in the "ground truth cell type" axis. Are the axes swapped or are they correct? Could you please clarify?
- In Figure 2E, what does the color indicate? Is it the power argument "n^x" or something else?
- The reader may be wondering:
  - How many query cells did you use?
  - How many clusters were in the query dataset? How many cells per cluster?
  - How many reference datasets were used?
  - How many clusters were in the reference dataset?
  - Were the query and reference datasets acquired from the same tissue sample or were they completely independent and unrelated?

In the section "Subclustering", please consider adding more details to help the reader avoid misunderstandings. What exactly is the "sub-clustering power argument (x)"? Please consider giving a concrete example to help the reader understand this section. Please consider creating a new figure that helps the reader to understand the "subcluster()" functionality.

 What is the PBMCbench data? Is this the same data as mentioned in the section "Correlation minimum cutoff"? 

 In the section "Cells per cluster", you might consider introducing the dataset, then introducing the question that is being addressed, and finally reporting the results. What is the number (15, 8, 4)? Is the "Mouse Cell Atlas" the same as the "Tabula Muris"? Were these mouse datasets used in the previous sections? The reader might benefit from an introduction of these datasets.

**Suggested improvements for Figure 3:**

- Please consider adding labels "A", "B", "C", "D" to mark each of the four panels, so they can be referenced clearly.
- Please consider using the same name consistently in the text and the figure titles. For example, the figure says "Bulk RNA-seq reference data" but the text says "ImmGen database". The reader might better understand the results if the same label were used in both places instead of using two different labels for the same thing.
- Please consider including the identifiers for readers who wish to find these datasets and download them. For example, if the datasets are available on NCBI GEO, please consider including the accession numbers directly in the legend text, or in a table. Check to see if any other database provides an accession number. If an accession number is not available, please consider providing the DOI for a publication or a URL for a website that provides the data. By the way, if any data you are using is not deposited to a permanent repository, please consider uploading this data to a permanent repository (e.g. Figshare).

In the section describing Figure 4A, please consider these suggested changes:

- Please explain what is "clustifyr", "clustifyr_lists", and "clustifyr_m3drop".
- How was feature selection performed for each analysis in Figure 4A?
- What is the strategy used by scmap?
- What is the strategy used by "Seurat"?
- What is the strategy used by "SingleR"?
- How is clustifyr similar or different?

This section says "Correlation-based clustifyr classification performed better than hypergeometirc-based gene list enrichment as implemented in clustify_lists." Please consider explaining the "clustify_lists" algorithm in detail and also consider sharing the quantification of the performance of each approach so the reader can interpret the claim "performed better". Also consider elaborating on "performed better".

 What is "scRNAseq_Benchmark subsampling"? Could you elaborate on what this is and why it was used?

**Suggested improvements for Figure 4:**

- Please consider including an overview schematic to help the reader understand which datasets were used for each result.
- Please define "accuracy". What is the algorithm for computing this number?
- Please define "medF1-score". What is the algorithm for computing this number?
- For the lower half of panel B, please consider using a format similar to the one in Figure 2B from Kiselev *et al.* (2018 ^1^). For example, please use a log10 axis for time, so readers can see the difference between methods.
- Why is "medF1-score" used for Figure 4C and "accuracy" for Figure 4B?

Why does Figure 4A have 6 methods, Figure 4B have 5 methods, and Figure 4C have 3 methods? Is it possible to include all 6 methods for all panels? Could you please comment on the reasons for excluding or including methods in each analysis?

- *"As we and others observe ^25^, novel algorithms may not be necessary for cell type classification, at least within the current limitations of sequencing technology and our broadstroke understanding of cell “types”. Rather, the generation of community curated reference databases is likely to be critical for reproducible annotation of cell types in scRNA-seq datasets."*

I agree that a community curated reference database would be a valuable contribution to the field. You might consider creating a table or other type of descriptive listing that helps the reader to understand all of the references that were used in this article. Consider including tissue source, healthy or disease status, number of cells and genes, technology used for the assay, DOI, data URL, NCBI GEO accession, or any other details that the reader might find helpful.

 Thank you for providing a GitHub repository with data files! Please also consider sharing the same data in compressed plain text format (e.g. "file.tsv.gz"). In addition to GitHub, please consider using a specialty service that is funded for the purpose of permanently archiving research data such as NIH Figshare ( https://nih.figshare.com). There are other options (Zenodo, Open Science Framework OSF, etc.).

- *"As an alternative, clustifyr also supports per-cell annotation, however the runtime is greatly increased and the accuracy of the cell type classifications are decreased due to the sparsity of scRNA-seq datasets, and requires a consensus aggregation step across multiple cells to obtain reliable cell type annotations."*

You might consider offering another alternative option. One extreme is to use the cluster averages, while the other extreme is to use single cells. Perhaps there might be a middle ground where clustifyr could automatically use k-means or some other algorithm to form clusters within the user-defined clusters. This would give the user even more flexibility.

 After reviewing the code, I can see that there is an "overcluster()" function that seems to do exactly what I suggested. Please consider describing this in the article and showing an example of how it works. In retrospect, I can see that the section titled "Subclustering" was supposed to describe this topic — I misunderstood this section on the first read.

 You may want to double-check all of the links in all of your HTML pages. I see three URLs:

- https://github.com/rnabioco/clustifyrdatahub/
- https://github.com/rnabioco/clustifyr
- https://github.com/rnabioco/clustifyrdata

I can see that the "clustifyrdatahub" repo has code for creating ".rda" files from the reference datasets.

 I also see similar scripts at https://github.com/rnabioco/clustifyrdata/tree/master/data-raw

 Readers might be confused when they see two different repos with similar scripts. You might consider deleting the "clustifyrdatahub" repo if it is not necessary.

 I'm happy to see that the data is organized and annotated in the GitHub repo. Specifically, in the GitHub "clustifyrdata" repo, in the "README.md" file, the table shows the name of the reference, the number of cell types, the number of genes, the organism, and a link to the publication. Please consider adding some version of this table to the article, so the reader can understand the scope of this article.

 After reviewing the code, I was able to resolve some of my misunderstandings caused by lack of clarity in the terse descriptions in this article. To reduce the chance of misunderstanding by other readers, you might consider clarifying or adding details to the descriptions of functions and results. For example, the article does not mention that GSEA is used to work with gene lists.

Are the conclusions about the tool and its performance adequately supported by the findings presented in the article?

Partly

Is the rationale for developing the new software tool clearly explained?

Yes

Is the description of the software tool technically sound?

Yes

Are sufficient details of the code, methods and analysis (if applicable) provided to allow replication of the software development and its use by others?

Partly

Is sufficient information provided to allow interpretation of the expected output datasets and any results generated using the tool?

Yes

Reviewer Expertise:

Bioinformatics, computational biology, immunogenomics, scRNA-seq.

I confirm that I have read this submission and believe that I have an appropriate level of expertise to confirm that it is of an acceptable scientific standard, however I have significant reservations, as outlined above.

## References

[1] : scmap: projection of single-cell RNA-seq data across data sets. *Nat Methods*.15(5) : 10.1038/nmeth.4644 359-362 10.1038/nmeth.4644 29608555

# Response

**Published:** 2020-07-07

**Authors:** Kent Riemondy

**Affiliations:** University of Colorado, Anschutz Medical Campus, USA

We thank the reviewer for their detailed suggestions which we believe have substantially improved the clarity of the manuscript. Our responses are indicated in italics below. 

The authors describe an R package for annotating cell clusters in scRNA-seq datasets. Specifically, the package implements code for computing correlations between the columns of two data matrices. They show that high correlations between unknown cell clusters in the first data matrix and annotated cell types in the second matrix can be used to label the unknown cell clusters. They try varying parameters and show the effects on the results, and they also benchmark the time and accuracy compared to other packages designed to annotate scRNA-seq data.

Details of the code, methods, and analyses are partly provided. Some details seem to be missing (e.g. the functionality for gene lists).

The conclusions about the tool and its performance are partly supported by the findings presented in the article. Some terms such as "medF1-score" and "accuracy" are left undefined, and some results omit some methods (Figure 4A has different methods than B or C). Readers may have difficulty understanding the specific questions that were asked and what results are shown.

Main comments:

- The clarity of the manuscript can be increased by adding more verbose details about all analyses. Please consider expanding details about each question, the approach, the datasets used, and the results.

*In an effort to more clearly present clustifyr we have added additional details about each dataset, the questions posed by the analysis, and the conclusions from each analysis.  *

- Please consider adding a table describing the reference datasets used in this article, just like the one shown on one of your GitHub repositories. This should help to summarize which datasets were used for the analyses in this article.

*We have added a table to the main text (Table 1) and a supplemental table that provide additional details about each dataset, and provide a reference of each dataset used in each figure panel.*

Comments about specific parts of the manuscript are below. Excerpts from the article are shown in "quoted italics" after a bullet point, and my comments are shown directly below the bullet point.

 

- "A key challenge in scRNA-seq data analysis is the identification of cell types from single-cell transcriptomes. Manual inspection of the expression patterns from a small number of marker genes is still standard practice, which is cumbersome and frequently inaccurate."

Do we know the accuracy by manual inspection? Is there a reference for this? In the absence of evidence, you might consider weakening the statement to say "may be inaccurate" rather than "is cumbersome and frequently inaccurate". You might consider that many scRNA-seq experiments are done for the purpose of discovering new cell types that have not been well-described in previous published datasets. In this setting, manual inspection is necessary and automated analyses could be inaccurate or misleading.

*To our knowledge there has not been a direct study of the accuracy of manual inspection compared to automated methods. We thank the reviewer for noting this point and have weakened this statement accordingly. We also have noted that automated methods can supplement manual inspection of markers to provide additional justification of the discovery of novel cell types. *

- "Currently, multiple cell type assignment packages exist but they are specifically tailored towards input types or workflows8–13."

Please consider naming and describing each method that will be compared to clustifyr in this manuscript, so the reader can assess how the methodology of clustifyr compares to other methods. Which methods are "specifically tailored towards input types or workflows"? Could you give an example to help the reader understand this claim?

*We have added descriptions of the methodologies used by tools that we compared clustifyr against (see Introduction). We also have noted which tools are tailored towards input types: reference single cell data (Seurat, ACTINN, scPred) or workflows: using Seurat objects  (Seurat), using singleCellExperiment (singleR, scPred), or using the command-line (ACTINN).*

Suggested improvements for Figure 1:

- In Figure 1, you might consider showing the dimensions of the inputs and outputs. This might help the reader to understand how they relate to each other.

*We have added the dimensions to provide clarity.*

- Should the query and reference data be counts? CPM? Or Log2(CPM + 1)? You might consider elaborating on this.

*Clustifyr supports both raw counts or log normalized values. The decision of which to use is left to the user and we recommend using similar normalization as used for the reference matrix, if possible. We have added text (under Variable gene selection and normalization) to provide guidance to the use*r.

Suggested improvements for Figure 2:

- Please consider rotating Figure 2A, D, and E 90 degrees clockwise to improve legibility.

*We have amended the figures as suggested.*

- Please consider limiting the axes ranges to the data instead of using the range [0, 1].

*We respectfully decline to implement this suggestion, as we believe restricting the plot to only the range of the data can over-emphasize minor differences in distributions.* 

- Please consider increasing all font sizes in all panels in all figures, including titles, legends, axis text, etc. Some readers might need larger sizes to see clearly.

*We have increased the font sizes accordingly.*

- Please consider changing the title to "All genes (n = 10,000)" and "M3Drop variable genes (n = 1,000)"  in Figure 2C, so we have some sense of the number of genes used to generate each heatmap.

*We have changed these titles as suggested.*

- Please consider showing a graphical representation of the experiment setup for this figure. What is the reference? What is the query? What are their dimensions? What is the main question in this analysis?

*We have added additional details about the query and reference datasets in the main text, legends, and in the titles of figure panels as an alternative to graphical representations. The questions addressed by each analysis are more clearly stated when introducing each figure panel. We believe that these edits now provide sufficient clarity for the reader to understand the content of each figure.*

- One way to enhance clarity is to add descriptive titles to every figure in every panel (e.g. "Testing different correlation statistics", etc.).

*We thank the reviewer for this suggestion and we have added titles to figure panels that we believe were unclearly presented.*

- Please consider adding more details to the legend text for Figure 2 to help readers understand exactly what experiment has been done, what data was used, and what result is shown.

*We have added additional details about each panel to the legend text as well as additional text to the main manuscript as requested.*

- In Figure 2C, it seems that the y-axis and x-axis have been swapped by mistake. I see that the y-axis is labeled "ground truth cell type" but it includes "unclassified". I would expect the category "unclassified" to appear in the "called cell type" axis, but not in the "ground truth cell type" axis. Are the axes swapped or are they correct? Could you please clarify?

*The unclassified cell type was annotated as unclassified in the original study, whereas the query dataset contained a cell type (schwann cells) that appears to be similar to the reference data “unclassified”.  We have added additional text to the legend to clarify.*

- In Figure 2E, what does the color indicate? Is it the power argument "n^x" or something else?

*The color did not indicate any particular class and therefore was removed. *

- The reader may be wondering:
  - How many query cells did you use?
  - How many clusters were in the query dataset? How many cells per cluster?
  - How many reference datasets were used?
  - How many clusters were in the reference dataset?
  - Were the query and reference datasets acquired from the same tissue sample or were they completely independent and unrelated?

*We have added a supplemental table (supplemental table 1) with additional details for each dataset in the manuscript. The tissues samples used for the query and reference datasets were derived from unrelated individuals or mice based on our reading of the original publications for each dataset. An exception was the PBMC-bench dataset, in which multiple single cell technologies were tested using the same aliquot of PBMCs. We have added text to the results section to clarify (under Correlation method).*

In the section "Subclustering", please consider adding more details to help the reader avoid misunderstandings. What exactly is the "sub-clustering power argument (x)"? Please consider giving a concrete example to help the reader understand this section. Please consider creating a new figure that helps the reader to understand the "subcluster()" functionality.

*We have added an additional figure (2E) to demonstrate the utility of the subcluster/overcluster_test functionality.*

What is the PBMCbench data? Is this the same data as mentioned in the section "Correlation minimum cutoff"? 

*Yes this is the same dataset. We have added additional details to the text to introduce this dataset, as well as additional details provided in table 1.* 

In the section "Cells per cluster", you might consider introducing the dataset, then introducing the question that is being addressed, and finally reporting the results. What is the number (15, 8, 4)? Is the "Mouse Cell Atlas" the same as the "Tabula Muris"? Were these mouse datasets used in the previous sections? The reader might benefit from an introduction of these datasets.

*We have provided additional text to the manuscript to introduce and describe these datasets to improve clarity about the analyses conducted. The x axis refers to the number of cells per cluster.*

Suggested improvements for Figure 3:

- Please consider adding labels "A", "B", "C", "D" to mark each of the four panels, so they can be referenced clearly.

*We have added these labels and referenced them in the updated figure legend.* 

- Please consider using the same name consistently in the text and the figure titles. For example, the figure says "Bulk RNA-seq reference data" but the text says "ImmGen database". The reader might better understand the results if the same label were used in both places instead of using two different labels for the same thing.

*We have added subtitles to each panel to more clearly reference the datasets in the text. *

- Please consider including the identifiers for readers who wish to find these datasets and download them. For example, if the datasets are available on NCBI GEO, please consider including the accession numbers directly in the legend text, or in a table. Check to see if any other database provides an accession number. If an accession number is not available, please consider providing the DOI for a publication or a URL for a website that provides the data. By the way, if any data you are using is not deposited to a permanent repository, please consider uploading this data to a permanent repository (e.g. Figshare).

*The publicly available datasets are referenced in the Data Availability section, with additional details now provided in Table 1. GEO accession numbers, DOIs, or URLs are provided, depending on the datasource. Additionally to further ease access to these resources we have organized these datasets into an ExperimentHub (clustifyrdatahub)  that is in the process of being submitted to bioconductor.*

In the section describing Figure 4A, please consider these suggested changes:

- Please explain what is "clustifyr", "clustifyr_lists", and "clustifyr_m3drop".
- How was feature selection performed for each analysis in Figure 4A?
- What is the strategy used by scmap?
- What is the strategy used by "Seurat"?
- What is the strategy used by "SingleR"?
- How is clustifyr similar or different?

*We have added an additional paragraph to explain the differing clustifyr methods shown in Figure 4A.  Feature selection was performed by the Tabula muris investigators using the variable genes selected by Seurat by examining a plot of the gene expression mean vs. variance (mean.var.plot). Seurat and clustifyr use these variable genes, whereas SingleR and scmap define variable genes using differential expression testing or M3Drop respectively. We have added text to explain the feature selection methods used by each benchmarked method.*

This section says "Correlation-based clustifyr classification performed better than hypergeometirc-based gene list enrichment as implemented in clustify_lists." Please consider explaining the "clustify_lists" algorithm in detail and also consider sharing the quantification of the performance of each approach so the reader can interpret the claim "performed better". Also consider elaborating on "performed better".

*We have elaborated on the clustifyr_lists approach for classifying cell types based on gene set enrichment in the text. Additionally we have included a comparison of two approaches that performed best in our benchmarking, using hypergeometric tests, or using the jaccard index and selecting the cell type with the highest index value (Figure 4A).*

What is "scRNAseq_Benchmark subsampling"? Could you elaborate on what this is and why it was used?

*We have added additional text to the result section to introduce this dataset. This dataset contains random subsets of the tabula muris dataset to enable investigation of performance and accuracy with varying cell numbers.*

Suggested improvements for Figure 4:

- Please consider including an overview schematic to help the reader understand which datasets were used for each result.

*We have added descriptive titles and additional text to the results section to describe the datasets and goals of each benchmarking test. *

- Please define "accuracy". What is the algorithm for computing this number?

*Accuracy is defined as the ratio between the number of correctly classified clusters and the overall number of clusters for every dataset pair.*

- Please define "medF1-score". What is the algorithm for computing this number?

*medF1-score was a shortened term for median F1-score. We have removed all references to medF1-score and replaced with median F1-score. F1-score, the harmonic mean of the precision and recall, is calculated for each cell type. A median F1-score is reported for every dataset pair.*

- For the lower half of panel B, please consider using a format similar to the one in Figure 2B from Kiselev et al. (2018 1). For example, please use a log10 axis for time, so readers can see the difference between methods.

*We have modified the figure accordingly.*

- Why is "medF1-score" used for Figure 4C and "accuracy" for Figure 4B?

*A F1-score cannot be calculated when the query and reference datasets contain different cell types. Therefore when comparing datasets with varying cell composition we instead utilized an accuracy metric (as defined above). We have added text to the manuscript that defines accuracy and median F1-score (Benchmarking methods), identifies which datasets were compared with each metric, and provides an explanation of why certain datasets were characterized with accuracy or F1-score.*

Why does Figure 4A have 6 methods, Figure 4B have 5 methods, and Figure 4C have 3 methods? Is it possible to include all 6 methods for all panels? Could you please comment on the reasons for excluding or including methods in each analysis?

*We agree that it is confusing for differing tools to be shown in different panels. We have therefore benchmarked these methods in a more consistent fashion to enable comparison of each method across different benchmarking tests. One exception however is scPred, which we were unable to successfully run on the Allen Brain Institute atlas data (Figure 4C), which we have noted in the figure legend.*

- "As we and others observe25, novel algorithms may not be necessary for cell type classification, at least within the current limitations of sequencing technology and our broadstroke understanding of cell “types”. Rather, the generation of community curated reference databases is likely to be critical for reproducible annotation of cell types in scRNA-seq datasets."

I agree that a community curated reference database would be a valuable contribution to the field. You might consider creating a table or other type of descriptive listing that helps the reader to understand all of the references that were used in this article. Consider including tissue source, healthy or disease status, number of cells and genes, technology used for the assay, DOI, data URL, NCBI GEO accession, or any other details that the reader might find helpful.

*In addition to the dataset details provided in the Data Availability section and the details provided in Table 1, we have also included a supplemental table that references the datasets used in each figure, and an ExperimentHub package allowing direct access to these resources in R.*

Thank you for providing a GitHub repository with data files! Please also consider sharing the same data in compressed plain text format (e.g. "file.tsv.gz"). In addition to GitHub, please consider using a specialty service that is funded for the purpose of permanently archiving research data such as NIH Figshare ( https://nih.figshare.com). There are other options (Zenodo, Open Science Framework OSF, etc.).

*The datasets used in this study were all published by other research groups and are hosted in various data repositories including GEO and figshare. As mentioned above we have provided additional methods to access these published and publicly available resources. *

- "As an alternative, clustifyr also supports per-cell annotation, however the runtime is greatly increased and the accuracy of the cell type classifications are decreased due to the sparsity of scRNA-seq datasets, and requires a consensus aggregation step across multiple cells to obtain reliable cell type annotations."

You might consider offering another alternative option. One extreme is to use the cluster averages, while the other extreme is to use single cells. Perhaps there might be a middle ground where clustifyr could automatically use k-means or some other algorithm to form clusters within the user-defined clusters. This would give the user even more flexibility.

After reviewing the code, I can see that there is an "overcluster()" function that seems to do exactly what I suggested. Please consider describing this in the article and showing an example of how it works. In retrospect, I can see that the section titled "Subclustering" was supposed to describe this topic — I misunderstood this section on the first read.

*We have added an additional figure panel (Figure 2E) to illustrate this functionality.*

You may want to double-check all of the links in all of your HTML pages. I see three URLs:

- https://github.com/rnabioco/clustifyrdatahub/
- https://github.com/rnabioco/clustifyr
- https://github.com/rnabioco/clustifyrdata

I can see that the "clustifyrdatahub" repo has code for creating ".rda" files from the reference datasets.

I also see similar scripts at https://github.com/rnabioco/clustifyrdata/tree/master/data-raw

Readers might be confused when they see two different repos with similar scripts. You might consider deleting the "clustifyrdatahub" repo if it is not necessary.

*We apologize to the reviewer for the confusion of multiple data repositories. We have organized clustifyrdata into an ExperimentalHub Bioconductor package at the request of reviewer #1, resulting in overlapping content in the clustifyrdatahub repository. We have mentioned these differences in the documentation of these repositories and added text to the manuscript to point readers to the experimentHub package, which is currently being submitted to bioconductor.*

I'm happy to see that the data is organized and annotated in the GitHub repo. Specifically, in the GitHub "clustifyrdata" repo, in the "README.md" file, the table shows the name of the reference, the number of cell types, the number of genes, the organism, and a link to the publication. Please consider adding some version of this table to the article, so the reader can understand the scope of this article.

*We have added a table (Table 1) to the main manuscript that contains additional details about each dataset.*

After reviewing the code, I was able to resolve some of my misunderstandings caused by lack of clarity in the terse descriptions in this article. To reduce the chance of misunderstanding by other readers, you might consider clarifying or adding details to the descriptions of functions and results. For example, the article does not mention that GSEA is used to work with gene lists.

*We have added additional details about the gene list methods (including GSEA) to the article. clustify() and clustify_lists() are the most important functions implemented in clustifyr, which we believe are now sufficiently described in the revised manuscript. Additional package and function level documentation is provided at https://rnabioco.github.io/clustifyr/ , which we’ve now provided as a link in the software availability section.*

# Peer review: Reviewer response for version 1

**DOI:** 10.5256/f1000research.25358.r61913

**Published:** 2020-04-20

**Authors:** Keegan Korthauer

**Affiliations:** 1 BC Children's Hospital Research Institute, Vancouver, BC, Canada; 2 University of British Columbia, Vancouver, BC, Canada

The article introduces a user-friendly and inter-operable R package for cell-type assignment of single-cell RNA-sequencing data. As clearly stated by the authors, the method heavily relies on the (1) results of and (2) any assumptions made by the clustering algorithm applied to the query dataset. The method has potential to be widely useful given its flexibility to take input and give output from many different existing (and future) algorithms. Although the methods proposed are not novel (simple correlation metrics), the software serves to streamline one of the most common procedures in single-cell RNA-sequencing analysis. As detailed below I have some questions regarding the evaluation of the method compared to existing approaches, and a suggestion to more widely distribute the prebuilt references curated as part of the study.

 Major comments:

1. The 'unseen population rejection test' is an informative measure. However, it is not clear without going back to the scRNAseq_Benchmark (Abdelaal *et al.*, 2019 ^1^) how clustifyr's performance compares to other tools. It would be useful to give some quantitative or visualization that conveys this comparison.
2. The approach is aimed at being "normalization-agnostic" as stated in 'Benchmarking' section. However, it's not clear whether this refers to clustifyr in general, or just using the rank correlation setting. If in general, this property should be demonstrated.
3. The benchmarking results provided are very helpful, but it's not clear why only a (differing) subset of the methods was applied to each evaluation (i.e. panels of Figure 4 in particular).

Minor comments:

1. From the description of the method, it seems that if the query dataset is 'over-clustered', meaning a cell-type is incorrectly split into two clusters, clustifyr can return the same cell type assignment for both clusters (provided the correct reference had the highest correlation, and that correlation was above the threshold). Is this correct? If not, please clarify.
2. The prebuilt references in the clustifyrdata github repository has potential utility to researchers who don't already have a reference dataset. It might be a good fit to build these reference datasets as a Bioconductor ExperimentHub package.

Are the conclusions about the tool and its performance adequately supported by the findings presented in the article?

Partly

Is the rationale for developing the new software tool clearly explained?

Yes

Is the description of the software tool technically sound?

Yes

Are sufficient details of the code, methods and analysis (if applicable) provided to allow replication of the software development and its use by others?

Yes

Is sufficient information provided to allow interpretation of the expected output datasets and any results generated using the tool?

Partly

Reviewer Expertise:

Statistical genomics, bioinformatics

I confirm that I have read this submission and believe that I have an appropriate level of expertise to confirm that it is of an acceptable scientific standard, however I have significant reservations, as outlined above.

## References

[1] : A comparison of automatic cell identification methods for single-cell RNA sequencing data. *Genome Biology*.2019;20(1) : 10.1186/s13059-019-1795-z 10.1186/s13059-019-1795-z 31500660PMC6734286

# Response

**Published:** 2020-07-07

**Authors:** Kent Riemondy

**Affiliations:** University of Colorado, Anschutz Medical Campus, USA

We thank the reviewer for their helpful criticisms. Our responses are indicated in italics below. 

Major comments:

- The 'unseen population rejection test' is an informative measure. However, it is not clear without going back to the scRNAseq_Benchmark (Abdelaal et al., 2019 1) how clustifyr's performance compares to other tools. It would be useful to give some quantitative or visualization that conveys this comparison.

*We agree and have provided an additional figure panel (4E) that provides a visual comparison of clustifyr’s performance compared to tools assessed by the scRNAseq_Benchmark. *

- The approach is aimed at being "normalization-agnostic" as stated in 'Benchmarking' section. However, it's not clear whether this refers to clustifyr in general, or just using the rank correlation setting. If in general, this property should be demonstrated.

*We are referring the property of rank correlation rather than a specific feature of clustifyr. We have amended the text (subsection: Variable gene selection and normalization) to make this point more clear and provide recommendations that users try to implement the same normalization scheme for reference and query data if possible.*


* *

- The benchmarking results provided are very helpful, but it's not clear why only a (differing) subset of the methods was applied to each evaluation (i.e. panels of Figure 4 in particular).

*We agree that the benchmarking would be more clearly presented by providing more complete assessment of methods across each evaluation. We have updated figures 4A,B,C, and D to consistently present clustifyr’s performance and accuracy compared to other methods. Of note, we were unable to benchmark scPred when examining the Allen Brain Atlas data ( Figure 4C), due to an error that we were unable to troubleshoot.*

Minor comments:

- From the description of the method, it seems that if the query dataset is 'over-clustered', meaning a cell-type is incorrectly split into two clusters, clustifyr can return the same cell type assignment for both clusters (provided the correct reference had the highest correlation, and that correlation was above the threshold). Is this correct? If not, please clarify.

*The reviewer's comment is correct, clustifyr will assign the cell type with the highest correlation, that meets a minimum cut-off value. For over-clustered query cell types, clustifyr will therefore return the same cell-type label, despite the overclustering. Clustifyr also provides a function (overcluster_test()) to intentionally overcluster the query dataset to potentially identify subpopulations that were grouped into another cell type due to inappropriate query dataset clustering. We have included an additional figure panel (2E) to illustrate this functionality.*

- The prebuilt references in the clustifyrdata github repository has potential utility to researchers who don't already have a reference dataset. It might be a good fit to build these reference datasets as a Bioconductor ExperimentHub package.

*We thank the reviewer for this suggestion and have built an ExperimentHub package that includes the prebuilt references in the clustifyrdata repository. The package (clustifyrdatahub) has been submitted to Bioconductor.*
